# Supplementary material for: Single Cell Transcriptomic and Chromatin Profiles Suggest Layer Vb Is the Only Layer With Shared Excitatory Cell Types in the Medial and Lateral Entorhinal Cortex
Source: Front Neural Circuits. 2022 Jan 26;15:806154. doi: 10.3389/fncir.2021.806154 (PMC8826650; doi:10.3389/fncir.2021.806154)
Supplement: Supplementary file 14 [file Data_Sheet_1.ZIP › LEC_Sup_vs_MEC_Sup_Motifs/homerResults/motif4.info.html]

Motif 4

## Information for 4-TGTTATCA (Motif 4)

A
C
G
T
A
C
T
G
G
A
C
T
A
G
C
T
C
G
T
A
A
C
G
T
A
G
T
C
C
G
T
A
  
Reverse Opposite:
